# Hyaluronic acid hydrogel encapsulated BMP-14-modified ADSCs accelerate cartilage defect repair in rabbits

**DOI:** 10.1186/s13018-021-02792-w

**Published:** 2021-11-03

**Authors:** Hao Liu, Yongjun Rui, Jun Liu, Fandong Gao, Yesheng Jin

**Affiliations:** Department of Traumatic Orthopedics, Wuxi Ninth People’s Hospital Affiliated To Soochow University, No. 999, Liangxi Road, Wuxi, 214000 Jiangsu Province People’s Republic of China

**Keywords:** BMP-14, Cartilage defect, HA hydrogel, ADSCs

## Abstract

**Background:**

Cartilage defect has a limited capacity to heal. In this context, we hypothesized that hyaluronic acid (HA) hydrogel encapsulated BMP-14-modified adipose-derived mesenchymal stem cells (ADSCs) could accelerate cartilage defect repair in rabbits.

**Methods:**

ADSCs were isolated and identified by flow cytometry. ADSCs were treated with adenovirus vector encoding BMP-14 (Ad-BMP-14) or adenovirus vector encoding control (Ad-ctrl). Real-time PCR (RT-PCR) and western blot assay was performed to verify the transfection efficacy and chondrogenic differentiation markers (ACAN, Collagen II and SOX9). Rabbit cartilage defect model was performed and randomly divided into following groups: control group, HA hydrogel + ADSCs, ADSCs, HA hydrogel + BMP-14 transfected ADSCs, HA hydrogel + BMP-14 transfected ADSCs. At 6, 9 and 12 weeks after surgery, scanning electron microscopy, hematoxylin–eosin, Safranin-O/Fast Green and immunohistochemical staining for Collagen II were performed to determine the role of HA hydrogel encapsulated BMP-14-modified ADSCs in cartilage repair in vivo.

**Results:**

ADSCs were successfully isolated and positively expressed CD29, CD44 and CD90. Transfection efficacy of Ad-BMP-14 was verified by RT-PCR and western blot assay. Moreover, Ad-BMP-14 could significantly increased chondrogenic differentiation markers (ACAN, Collagen II and SOX9). The LV-BMP-14-ADSCs and HA hydrogel + LV-BMP-14-ADSCs groups revealed smoother surface cartilage repair that was level with the surrounding cartilage and almost complete border integration.

**Conclusions:**

HA hydrogel encapsulated BMP-14-modified ADSCs accelerate cartilage defect repair in rabbits. We need to further validate the specific mechanism of action of HA hydrogel encapsulated LV-BMP-14-ADSCs involved in the repairing cartilage damage in vivo.

## Background

Articular cartilage defects are common disorders that affect people of all ages; treatment of this disorder remains challenging [1–3]. With annual costs of US $560–$635 billion annually, articular cartilage defects accounts for nearly 1–2.5% of GDP of the GDP [4, 5]. Thus, articular cartilage defects cause a large socioeconomic burden. Articular cartilage has a limited ability to regenerate following injury due to its low cellularity, lack of vascularization and the low proliferative and migratory capacities of chondrocytes [6].

In order to solve this dilemma, transplantation of cultured chondrocytes or mesenchymal stem cells (MSCs) can regenerate cartilage tissue in cartilage defects [7]. There are multiple sources of MSCs including the bone marrow, adipose tissues and the umbilical cord [8–11]. Adipose-derived mesenchymal stem cells (ADSCs) have attracted attention because they are easily accessible in abundant quantities [12]. ADSCs are capable of self-renewal and differentiation into cells such as osteoblasts, chondrocytes, and adipocytes [13]. ADSCs are readily differentiate into adipocytes when in vitro cultured [14].

Furthermore, gene transfection of chondrogenesis gene could facilitate the differentiation of MSC and, as a result, improve the cartilage repair in the defect area [15]. Bone morphogenetic protein-14 (BMP-14), also known as growth differentiation factor-5 (GDF-5), is a potent chondro-inductive cytokine [16]. BMP-14 influences endochondral bone growth and the formation of cartilage [17–19].

Therefore, by transfecting ADSCs with BMP-14 gene can persistently expressed of BMP-14 and thus facilitated ADSCs into chondrocytes. Moreover, transplanted BMP-14 gene modified ADSCs into the defected cartilage could promote cartilage defect repair.

## Material and methods

### Primary ADSCs culture and BMP-14 transfection

Rabbit subcutaneous fat were obtained from 8-week-old female New Zealand White rabbits. Fat tissues were then cut into cubes of about 0.5 cm^3^ and kept in PBS solution. The blood vessel and fascia were cleared with an ophthalmic scissors. Then, fat tissues were digested by collagenase type I (0.1%) at 37 °C for 60 min. Cell extracts were centrifuged at 500×*g* for 10 min. Cells were inoculated in a 25 cm^2^ cell culture flask with a density of 2 × 10^6^/mL. Cells of passage 3–5 were used for subsequent experiments.

Then, flow cytometry was used for identifying stem cell surface markers: CD29, CD44, and CD90 ADSCs. In brief, after ADSCs digestion and centrifugation, a single-cell suspension was prepared with serum culture medium. Then, the cell density was adjusted to the density of 10^6^ cells/mL. ADSCs were incubated human anti-CD29, CD44, and CD90 antibodies. After PBS was washed, PBS was added to suspend the cells and tested on the computer.

Briefly, ADSCs at passage 3 were seeded into 6-well cell culture plates at 2 × 10^5^/ml. Culture medium was replaced with serum-free medium when the cells reached 80–90% confluence. Then, ADSCs were transfected with an adenovirus containing rabbit green fluorescent protein (GFP)-labeled BMP-14 (Ad-rBMP-14-GFP; 10^6^ PFU/ml) or an adenovirus containing GFP (Ad-ctrl-GFP; 10^6^ PFU/ml).

Recombinant adenoviruses were used to infect ADSCs at a multiplicity of infection (MOI) of 100 according to the instructions. After 24, 48 and 72 h incubation, the result of cell transfection was observed under a fluorescence microscope.

### Reverse transcription polymerase chain reaction (RT-PCR)

The TRIzol method (Invitrogen, Carlsbad, CA, USA) was performed to extract the total cellular RNA. RNA was reverse transcribed to cDNA using a reverse transcription kit (Takara, Dalian, China) according to the manufacturer’s protocols. Quantitative real‐time PCR (qRT-PCR) was performed using a quantitative SYBR Green PCR Kit (Takara Bio, Takara, Dalian, China). The reaction system was prepared with pre-denaturation at 94 °C for 30 s, denaturation at 94 °C for 5 s, annealing at 60 °C for 15 s, and extension at 72 °C for 10 s, and 45 cycles were amplified. Results were quantified using the comparative threshold method. Gene expression was calculated according to the 2^−ΔΔCT^ method. GAPDH was employed as the internal control. RT-PCR was done three times. The primers were designed and synthesized by Guangzhou Ribo Technology Co., Ltd. Primers were as follows: Aggrecan, (F), 5′ ATGGCTTCCACCAGTGCG-3′; (R), 5′-CGGATGCCGTAGGTTCTCA-3′; Collagen II, (F), 5′-GCACCCATGGACATTGGAGG-3′; (R), 5′-AGCCCCGCACGGTCTTGCTT-3′; SOX9, (F), 5′-TCCCTGAGACCCTAACTTGTGA-3′; (R), 5′-AGTCTCAGGGTCCGAGGTATTC-3′; BMP-14: (F), 5′-TGCCACTGTTGAGTGCAAGTC-3′ and (R) 5′-ACCTGGAGAAGCCGAAGGTAA-3′, β-actin (F), 5′-GGAGTCTACTGGCGTCTTCAC-3; (R), 5′-ATGAGCCCTTCCACGATGC-3′.

### Western blot analysis

The ADSCs were washed by cold PBS 3 times, then 150 μL RIPA lysate (Beyotime Biotechnology, Shanghai, China) was added to extract total protein. The cells were lysed in ice water by ultrasound, and the protein content was determined by the BCA method (BCA Protein Assay Kit, Solarbio, Beijing, China). An equal amount of proteins were taken from each group for 10% SDS-PAGE, and the proteins on the gel were transferred to PVDF membranes (Millipore, Bedford, MA, USA). The membranes were blocked at 4 °C for 1 h and then incubated at 4 °C overnight with the following primary antibodies (concentration: 1:1000): the BMP-14 antibody (ab93855, Abcam, USA), anti-ACAN antibody (13880-1-AP, Proteintech, Wuhan, China), Anti-collagen II antibody (28459-1-AP, Proteintech, Wuhan, China), anti-SOX9 antibody (67439, Proteintech, Wuhan, China) and anti-β-actin antibody (66009, Proteintech, Wuhan, China). After being cleaned twice with TBST, the membranes were incubated at room temperature for 1 h with fluorescein-labeled Goat anti-Rabbit IgG (ab205718, 1:2000). The antibodies were all from Abcam (Cambridge, UK). Finally, the membranes were cleaned three times, exposed with the ECL chromogenic agent (Millipore, Bedford, MA, USA), and imaged with an automatic developer (ChemiDoc XRS imaging system).

### Animals

This study was approved by the laboratory animal ethics committee of Wuxi Ninth People’s Hospital affiliated to Soochow University Nantong University. Once the animal was satisfactory anesthesia, rabbits were placed in a supine position on the operating table with the upper and lower limbs extended and fixed to the table. We cut the skin layer by layer and medial para-patellar approach was used to expose the knee. Full-thickness cartilage defects (3.5 mm in diameter, 3.0 mm in depth) were created in the center of the trochlear groove using a drill. Then, the knees were randomly divided into following groups: control group, HA hydrogel + ADSCs, ADSCs, HA hydrogel + BMP-14 transfected ADSCs, BMP-14 transfected ADSCs and HA hydrogel.

### Scanning electron microscopy observation

Following double fixation with glutaraldehyde, dehydration with a graded ethanol series and coating with epoxy resin (Epon 812; Byxbio, Jiangsu, China), scanning electron microscopy was used to observe the morphology of Ad-mock-GFP- and Ad-hBMP7-GFP-transfected chondrocytes in Matrigel.

### Hematoxylin and eosin (H&E) staining

Hematoxylin and eosin (H&E) and Safranin O-fast green staining were employed to assess the degree of cartilage degeneration. The rabbits’ knee joints were immobilized, decalcified, paraffin-embedded, and cut into 5 μm continuous histological sections. Two consecutive sections from the middle were analyzed using H&E and Safranin O-fast green staining.

### Immunohistochemistry (IHC)

The sampling method is the same as that of Safranin O-Fast green staining. Articular cartilage tissues were immobilized in 4% paraformaldehyde and paraffin-embedded. The block was sectioned (5 μm), and slides were prepared. Then, slides were incubated with primary antibodies for Collagen II (dilution 1: 2 00, Abcam) at 4 °C overnight. The next day, slides were rinsed three times and incubated with polyclonal antibodies against rabbit IgG-HRP at room temperature for 1 h.

### Statistical analysis

The experimental data are presented as mean ± standard deviation. The statistical software package SPSS version 13.0 (SPSS, Inc., Chicago, IL, USA) was used for data analysis. One-way analysis of variance (one-way ANOVA) followed by Dunnett’s test was adopted for comparison between groups, where *P* < 0.05 was considered to indicate a statistically significant difference.

## Results

### ADSC culture and identification

**Fig. 1:**
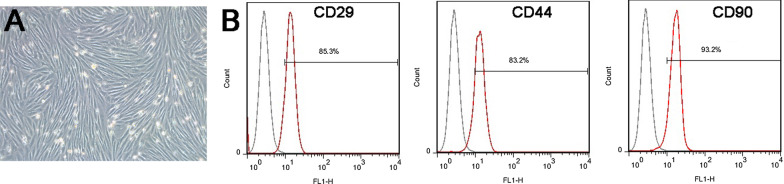
**A** Cell morphology of ADSCs at passage 3; **B** flow cytometry analysis showing that rBMSCs express CD29, CD44 and CD90

ADSCs were adherent and showed a typical spindle-like, long and elongated fibroblastic shape (Fig. 1A). Moreover, ADSCs strongly expressed CD29, CD44 and CD90, and its positive rate was 85.3%, 83.2% and 93.2% respectively (Fig. 1B).

**Fig. 2:**
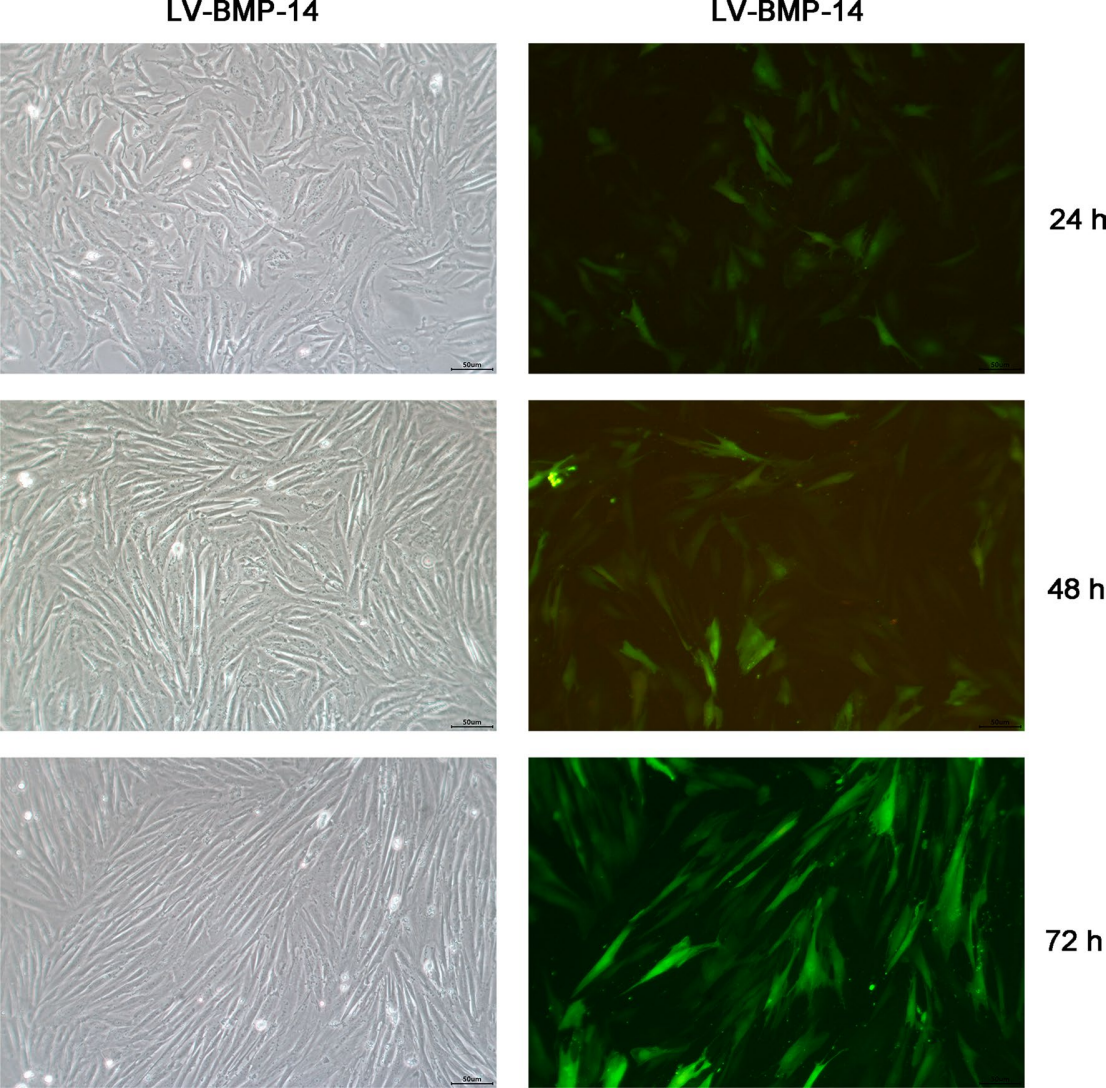
Green fluorescence protein (GFP)-expressing ADSCs shown by fluorescence microscopy at 24, 48 and 72 h after transfection

The fluorescence intensity of LV-BMP-14 in ADSCs increased along with the GFP fluorescence in a time-dependent manner (24 h, 48 h and 72 h). These results revealed that the transfection is successful (Fig. 2).

### BMP-14 overexpression increased chondrogenic differentiation markers

**Fig. 3:**
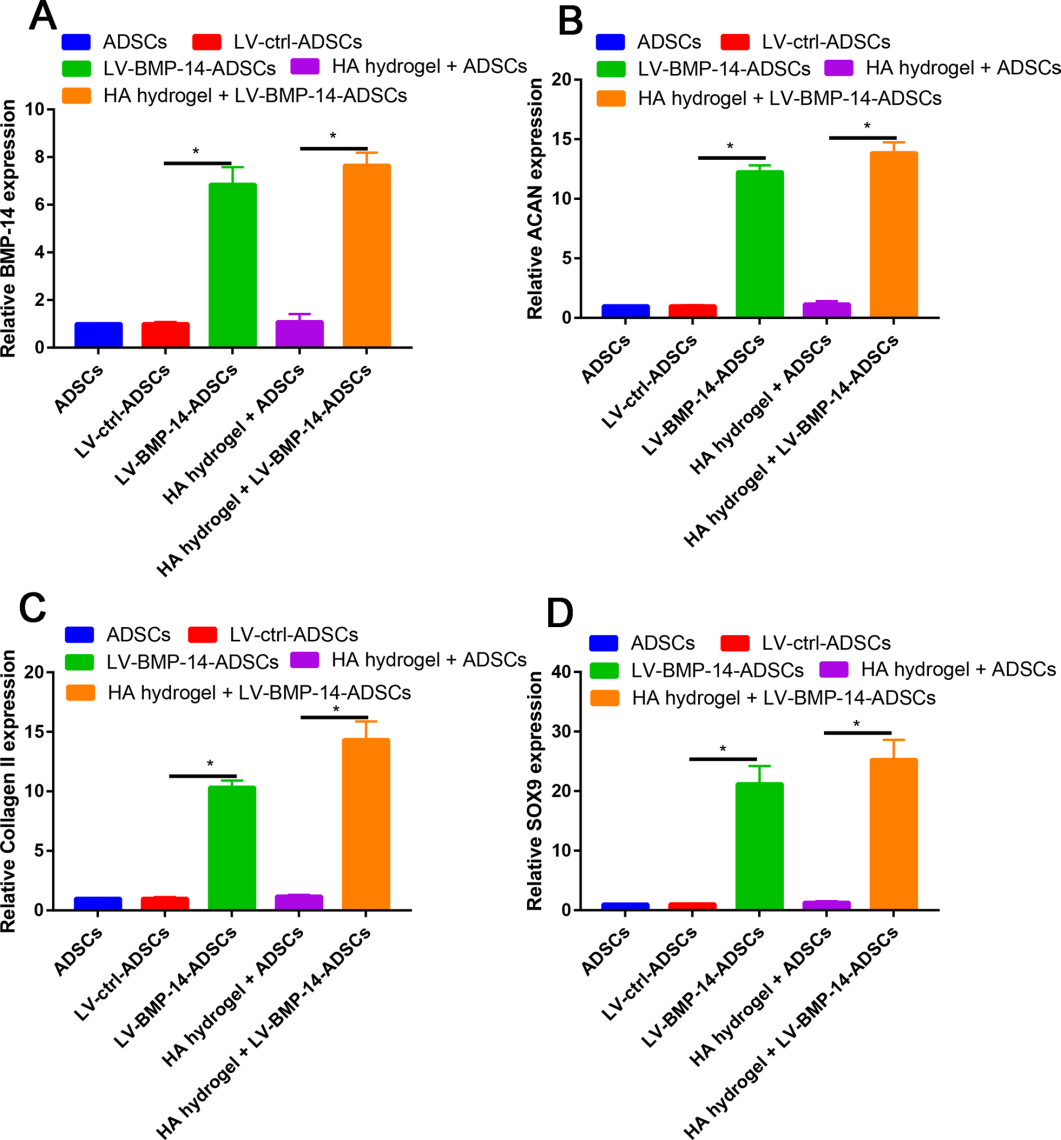
RT-PCR was performed to identify the BMP-14 (**A**), ACAN (**B**), Collagen II (**C**) and SOX9 (**D**) expression in ADSCs, LV-ctrl-ADSCs, LV-BMP-14-ADSCs, HA hydrogel + LV-ctrl-ADSCs, HA hydrogel + LV-BMP-14-ADSCs groups

**Fig. 4:**
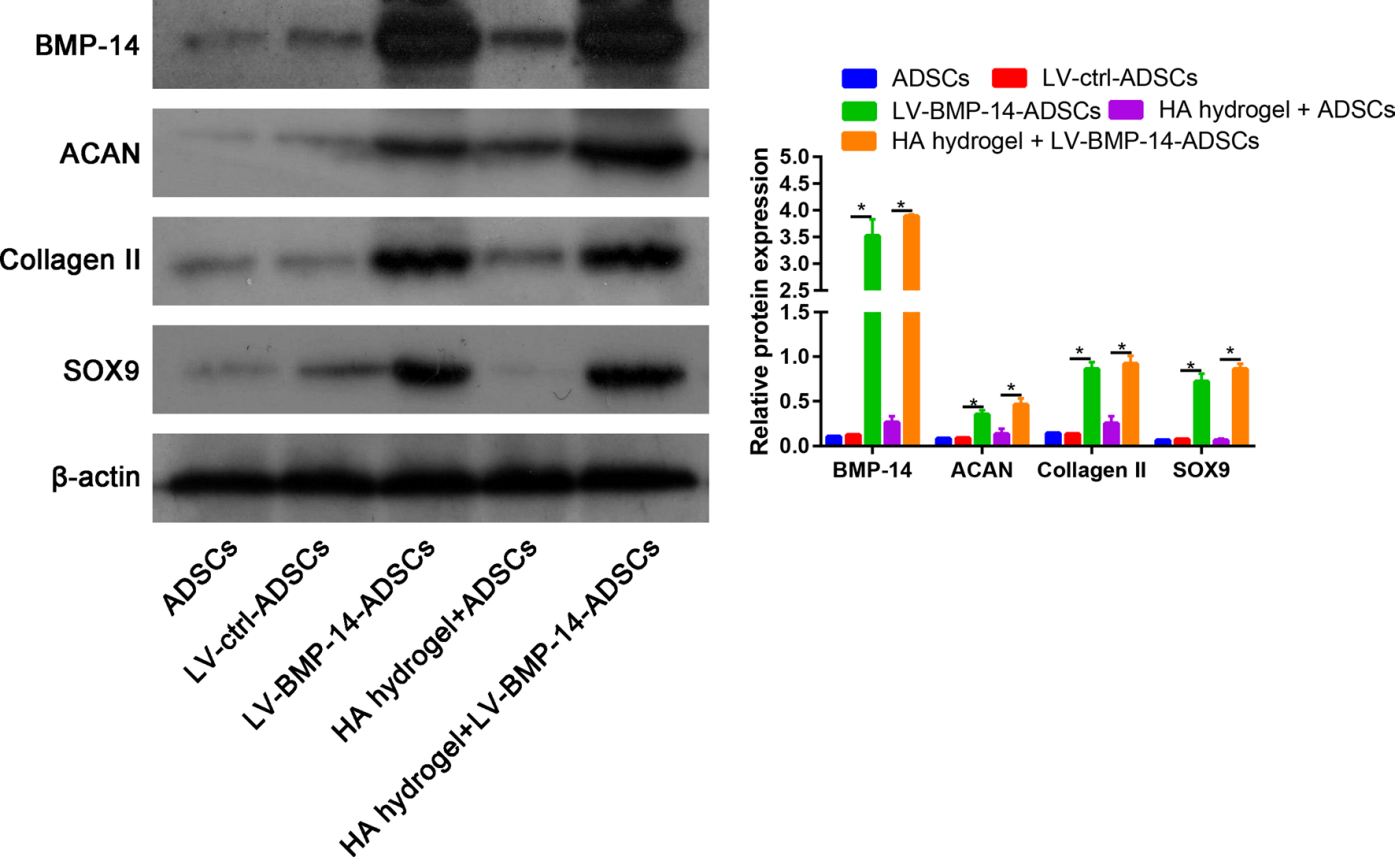
Western blot assay was performed to identify the BMP-14, ACAN, Collagen II and SOX9 expression in ADSCs, LV-ctrl-ADSCs, LV-BMP-14-ADSCs, HA hydrogel + LV-ctrl-ADSCs, HA hydrogel + LV-BMP-14-ADSCs groups

The successful transfection of BMP-14 was verified by quantitative real-time PCR (Fig. 3A). We found that BMP-14 expression was not alert between ADSCs or LV-ctrl-ADSCs groups. However, BMP-14 expression was significantly increased in LV-BMP-14-ADSCs or HA hydrogel + LV-BMP-14-ADSCs when compared with LV-ctrl-ADSCs or HA hydrogel + LV-ctrl-ADSCs respectively (Fig. 3A). Next, we performed PCR to identify the whether overexpression BMP-14 could enhance the chondrogenic differentiation of ADSCs. We found that after transfected with LV-BMP-14, the chondrogenic differentiation markers (ACAN, Collagen II and SOX9) was significantly increased than control group (Fig. 3B–D). Western blot analysis showed a result consistent with that in real-time PCR (Fig. 4).

### SEM

**Fig. 5:**
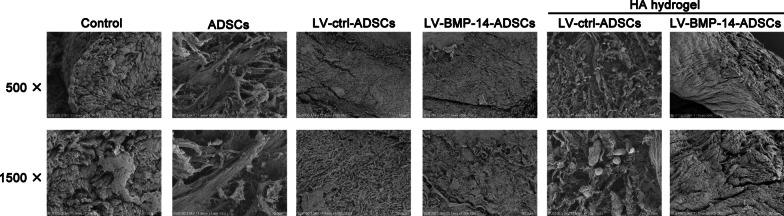
Evaluation of surface of cartilage at 12 weeks postoperatively by scanning electron microscopy (500 × and 1500 ×)

Those cartilage in the control group were significantly deteriorated with a rough appearance where the surface had fissures that had widened and collagen fibers that were exposed, loose, broken, and turned upwards. The rough appearance changes were partially improved by ADSCs or LV-ctrl-ADSCs treatment. In the LV-BMP-14-ADSCs group, the articular cartilage surface was no longer flat, with a visibly twisted wrinkled texture and a slightly concave shape. In BMP-14 transfected ADSCs and HA hydrogel group, cartilage exhibited similar to normal cartilage, in which a uniform area without splits, lacunae, or cartilage proliferation (Fig. 5).

### Histomorphometry of the repaired tissue

At 6, 9 and 12 weeks after cartilage defect surgery, the defect in the control group was filled with fibrous tissues, with a small number of cells attached and arranged irregularly.

**Fig. 6:**
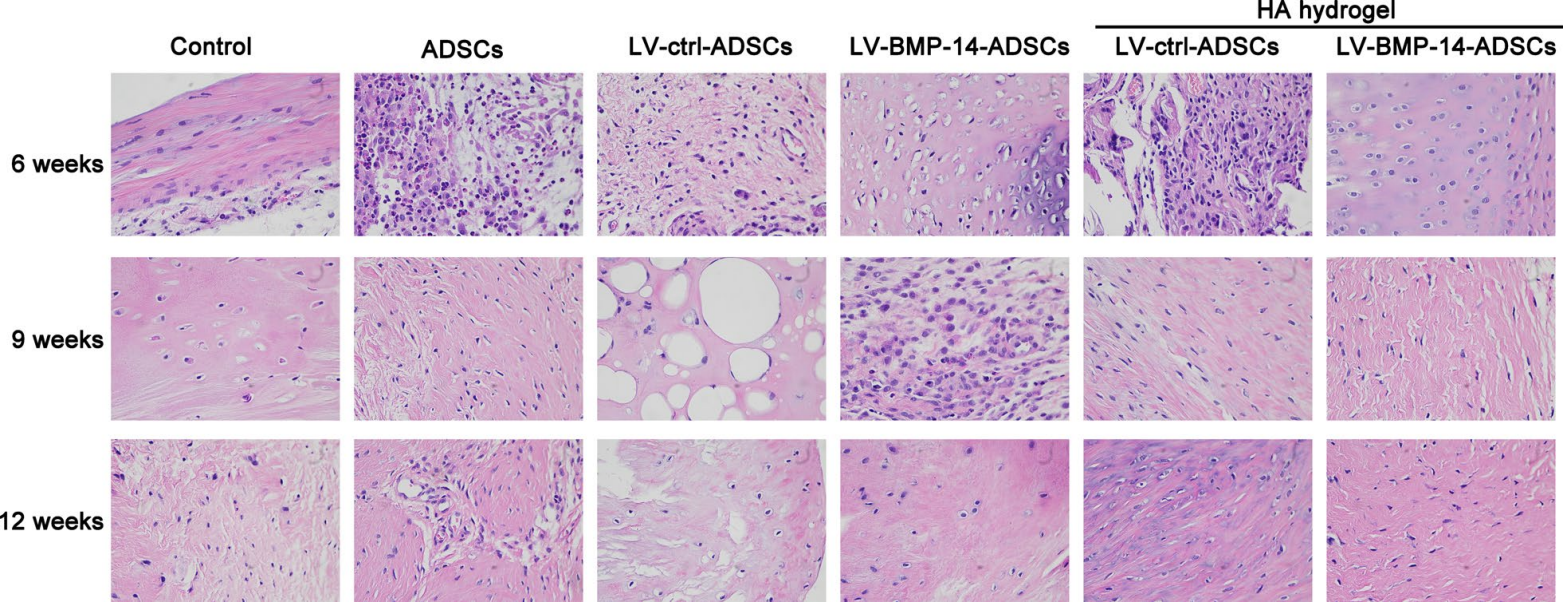
Histological evaluation of repaired tissue. Representative hematoxylin and eosin staining of repaired knees at 6, 9 and 12 weeks. Staining was repeated twice or more independently

In the ADSCs and LV-ctrl-ADSCs groups, the defect was covered with a thin cell coating arranged in order. Histomorphometry of the repaired tissue between the ADSCs and LV-ctrl-ADSCs groups did not differ. Furthermore, the LV-BMP-14-ADSCs and HA hydrogel + LV-BMP-14-ADSCs groups revealed smoother surface cartilage repair that was level with the surrounding cartilage and almost complete border integration (Fig. 6).

### Safranin O-fast green cartilage stain

**Fig. 7:**
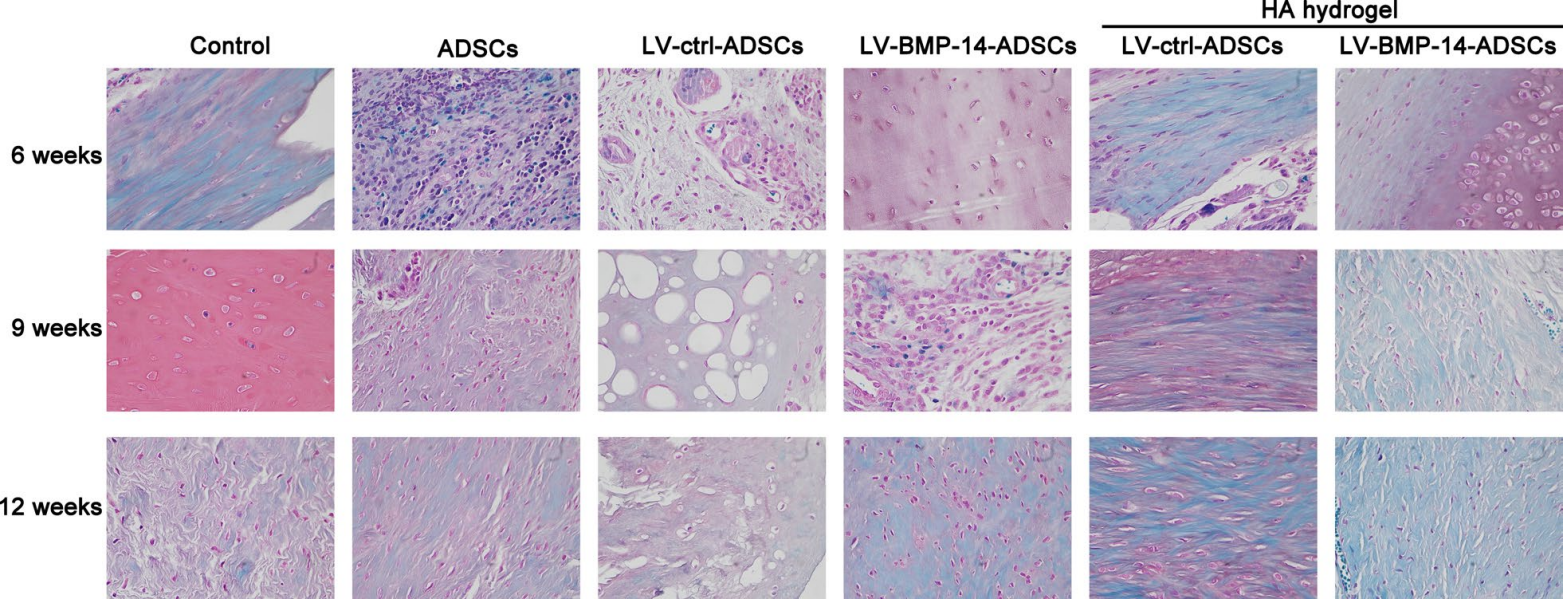
Histological evaluation of repaired tissue. Representative Safranin-O/Fast Green staining of repaired knees at 6, 9 and 12 weeks. Staining was repeated twice or more independently

Filling of the defect, Safranin O was used to identify the extracellular matrix. We found that reduced GAG deposition and irregular bone islands were observed in control group at 6, 9 and 12 weeks. In the control, ADSCs, LV-ctrl-ADSCs, LV-BMP-14-ADSCs, HA hydrogel + LV-ctrl-ADSCs and HA hydrogel + LV-BMP-14-ADSCs, the loss of articular cartilage increased in a time-dependent manner. In the ADSCs and LV-ctrl-ADSCs groups, the defect was repaired by fibrous tissue with an improved of the GAG content. Notably, the GAG differed among groups, with the significantly highest GAG was observed in the HA hydrogel + LV-BMP-14-ADSCs group relatively to ADSCs or LV-ctrl-ADSCs groups (Fig. 7).

### Collagen II staining

**Fig. 8:**
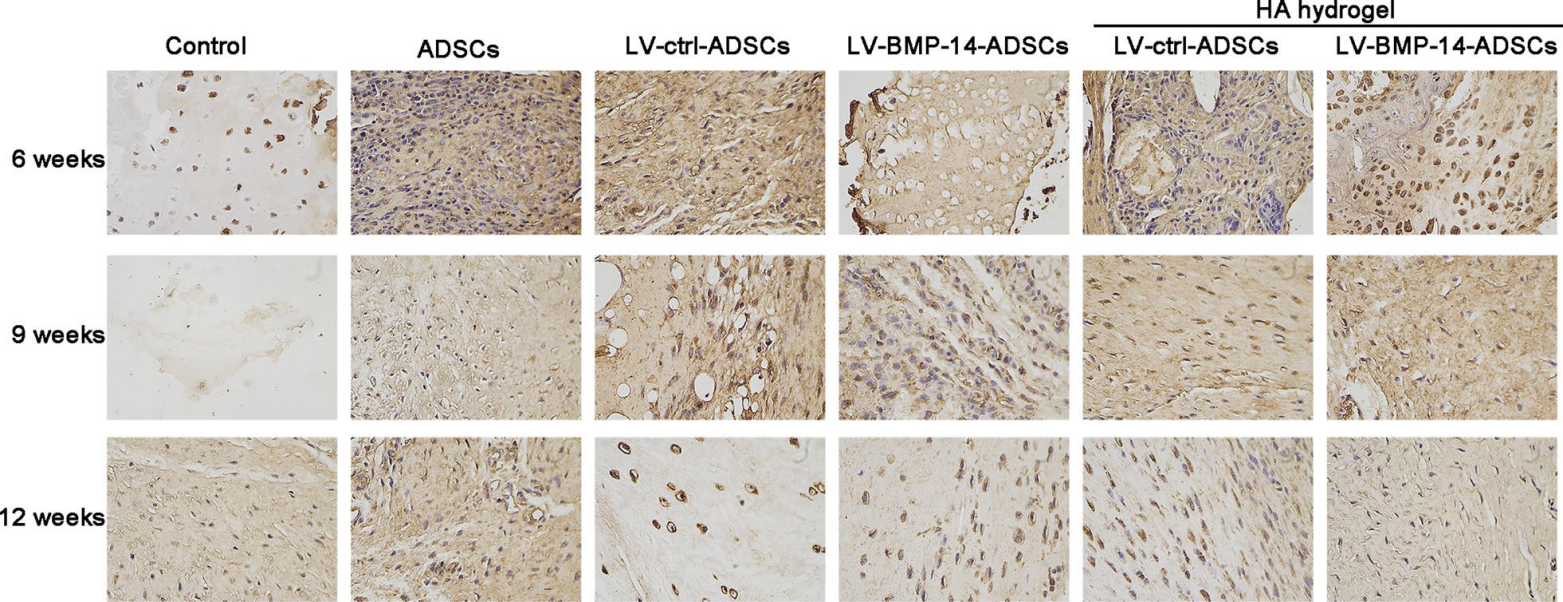
Immunohistochemical staining for Collagen II of repaired tissue. Representative Immunohistochemical staining for Collagen II of repaired knees at 6, 9 and 12 weeks. Staining was repeated twice or more independently

The defect region was mostly filled by fibrotic tissue with lower expression of type II collagen (Fig. 8). Excretion of collagen II increased with time at 6, 9 and 12 weeks, as indicated by collagen II staining. ADSCs or LV-ctrl-ADSCs treatment significantly increased the expression of collagen II when compared with the level in the control group at each time point (Fig. 8). Compared with ADSCs or LV-ctrl-ADSCs group, LV-BMP-14-ADSCs or HA hydrogel + LV-BMP-14-ADSCs significantly increased the collagen II expression (Fig. 8).

## Discussion

In this study, we constructed a LV-BMP-14-ADSCs for the first time. Moreover, we found that overexpression of BMP-14 could enhance the chondrogenic differentiation of ADSCs. We then used HA hydrogel encapsulated LV-BMP-14-ADSCs to repair cartilage defect. Results revealed that HA hydrogel combined with LV-BMP-14-ADSCs could promote cartilage defect repair.

The fact that ADSCs have been shown to be suitable seed cells for cartilage defect. Previous studies have identified that ADSCs could potentially serve as the experimental basis for a new treatment to repair cartilage defects in vivo. However, TGF-β was commonly used as a multifunctional factor that promote chondrogenic differentiation of ADSCs. However, TGF-β is more susceptible to inactivation and with a short half-life. These shortcomings of TGF-β limiting the clinical application of ADSCs for the treatment of cartilage defect. In the present study, the BMP-14 gene was stably transfected into ADSCs to induce its overexpression. PCR and western blot verified that BMP-14 was successfully transfected into ADSCs. Then, chondrogenic differentiation markers (ACAN, Collagen II and SOX9) was measured in different treatment groups. Results found that HA hydrogel encapsulated LV-BMP-14-ADSCs can enhance cartilage repair at the defect site.

BMP-14 is best known for its role in joint formation, epidermal stem cell proliferation, migration], apoptosis, and differentiation in vitro. Zhao et al. [20] found that BMP-14 promotes epidermal stem cells proliferation via Foxg1-cyclin D1 signaling.

Recently, BMP-14 also shown have an influence on endochondral bone growth. In contrast, BMP-14 deficiency significantly inhibited long bone fracture healing [19]. Buxton et al. [18] found that BMP-14 gene is expressed in the early cartilage condensation. Moreover, BMP-14 could also promotes the initial stages of chondrogenesis by promoting cell adhesion [18]. Luo et al. [21] found that adenovirus-mediated BMP-14 promotes the extracellular matrix expression in degenerative nucleus pulposus cells. Those results suggested that BMP-14 play an important role in chondrogenesis process of stem cells or nucleus pulposus cells. Moreover, BMP-14 could suppressed IL-1β induced inflammation and enhances TGF-β3-mediated chondrogenic differentiation in human rheumatoid fibroblast-like synoviocytes [22].

In this study, we found that BMP-14 overexpression significantly increased chondrogenic differentiation markers (ACAN, collagen II and SOX9).

Then, in vivo cartilage defect model was performed and then HA hydrogel encapsulated ADSCs or LV-BMP-14-ADSCs was used to assess the function of overexpression BMP-14 for repair of cartilage defect. We found that the HA hydrogel encapsulated LV-BMP-14-ADSCs had better cartilage repair than the control or ADSCs group. Moreover, HA hydrogel encapsulated LV-BMP-14-ADSCs was superior than LV-BMP-14-ADSCs alone. The reason may be that HA hydrogel can improve the effect of the ADSCs released. Previously, HA-derived hydrogels can deliver cells and therapeutic agents for tissue repair and regeneration [23]. Choi et al. [24] revealed that injectable basic fibroblast growth factor-loaded alginate/HA hydrogel induced successful glottal gap closure.

The important limitation in this paper is the dearth of large-animal models of cartilage defect (goats or pig). Another limitation of this study was lack of large-scale studies to validate the biosecurity safety of the BMP-14 gene transfer.

## Conclusion

Overall, our study testified that ADSCs with BMP-14 gene transfer by adenovirus could enhance chondrogenic differentiation of ADSCs. Moreover, HA hydrogel encapsulated LV-BMP-14-ADSCs considerably facilitated cartilage regeneration. This study demonstrates the potential of the HA hydrogel encapsulated LV-BMP-14-ADSCs in repairing cartilage defect and regenerating cartilage tissue. However, we need to further validate the specific mechanism of action of HA hydrogel encapsulated LV-BMP-14-ADSCs involved in the repairing cartilage damage in vivo.

## Data Availability

We state that the data will not be shared since all the raw data are present in the figures included in the article.

## Abbreviations

HA: Hyaluronic acid
ADSCs: Adipose-derived mesenchymal stem cells
Ad-BMP-14: Adenovirus vector encoding BMP-14
Ad-ctrl: Adenovirus vector encoding control
RT-PCR: Real-time PCR
SEM: Scanning electron microscopy
HE: Hematoxylin–eosin
MSCs: Mesenchymal stem cells
BMP-14: Bone morphogenetic protein-14
GDF-5: Growth differentiation factor-5
MOI: Multiplicity of infection
IHC: Immunohistochemistry
